# The Reported Use of Nosebands in Racing and Equestrian Pursuits

**DOI:** 10.3390/ani10050776

**Published:** 2020-04-30

**Authors:** Dominic Weller, Samantha Franklin, Glenn Shea, Peter White, Kate Fenner, Bethany Wilson, Cristina Wilkins, Paul McGreevy

**Affiliations:** 1 Sydney School of Veterinary Science, Faculty of Science, University of Sydney, Sydney NSW 2006, Australia; glenn.shea@sydney.edu.au (G.S.); p.white@sydney.edu.au (P.W.); kate@kandooequine.com.au (K.F.); bethany.wilson@sydney.edu.au (B.W.); paul.mcgreevy@sydney.edu.au (P.M.); 2 School of Animal and Veterinary Sciences, University of Adelaide, Roseworthy Campus, Roseworthy SA 5371, Australia; sam.franklin@adelaide.edu.au; 3 Saddletops Pty Ltd, PO Box 557, Gatton, Queensland 4343, Australia; editor@horsesandpeople.com.au

**Keywords:** horse, equitation science, welfare, safety

## Abstract

**Simple Summary:**

Nosebands are commonly used in many equestrian and racing disciplines. Despite common industry knowledge regarding the correct adjustment of nosebands, there seems to be a trend of overtightening nosebands and exposing horses to high pressures that restrict normal behaviours. Thus, there are concerns that nosebands could have harmful physical and behavioural impacts on horses. This article reports the results of an online survey of horse owners, riders and trainers that explored the distribution of common noseband designs across various disciplines, the reasons for their use, their perceived effectiveness, design preferences and how tightness is monitored, as well as detrimental consequences of their use. Most respondents reported using Plain Cavesson nosebands, with Hanoverian nosebands and so-called “cranking” systems also being common. Reasons for using nosebands varied widely among respondents according to noseband type and discipline. Preventing a horse’s tongue from moving over the bit, improving its appearance and aligning with the rules of the sport were the most nominated options. Almost a fifth of respondents reported physical and behavioural complications related to noseband use. The most common complication was hair loss under the noseband. Most respondents specified that they check noseband tightness at the bridge of the nose. Given the emerging discourse around restrictive nosebands and horse welfare, this article can inform industry and regulatory bodies about the types of nosebands used on horses in training and competition, the reasons for using nosebands and how noseband tightness is being monitored.

**Abstract:**

This article reports on the results of a survey designed to explore the types of nosebands that owners, riders and trainers use in training and competition, their reasons for using nosebands, the design preferences in different disciplines and approaches to noseband tightness and monitoring, as well as the incidence of negative impacts related to noseband usage. Respondents (*n* = 3040) were asked to specify the type of noseband they were currently using and to rate how effective they were in achieving these stated reasons. Respondents who used nosebands (*n* = 2332) most commonly used Plain Cavesson (46.6%, *n* = 1087) and Hanoverian (24.8%, *n* = 579) nosebands. The reasons provided in the survey for noseband usage were grouped into three broad, mutually exclusive categories: Anatomical; Consequential and Passive. Responses across these categories were fairly evenly distributed overall: Anatomical (29.5%, *n* = 1501), Consequential (30.6%, *n* = 1560), Passive (32.9%, *n* = 1673) and other reasons (7.0%, *n* = 358). Across all respondents (*n* = 2332), the most common Anatomical reason given was to *prevent the horse’s tongue from moving over the bit* (20.8%, *n* = 485), the most common Consequential reason was to *improve the appearance of the horse* (20.4%, *n* = 476), with *aligning with the rules of the sport* (30.2%, *n* = 705) the most popular Passive reason. Of the respondents who answered the question of checking noseband tightness (*n* = 2295), most reported checking noseband tightness at the bridge of the nose (62.1%, *n* = 1426), some (10.4%, *n* = 238) reported checking for tightness on the side of the face and others under the chin (21.5%, *n* = 496). This survey also revealed some of the potential issues associated with noseband use, with 18.6% (*n* = 434) reporting at least one physical or behavioural complication. The most common complication was hair loss under the noseband (39.9%, *n* = 173). Crank systems were reported to be used by 28.9% (*n* = 665) of respondents. This is of concern as these devices can be excessively tightened, minimising jaw and tongue movement and may compromise horse welfare. Indeed, the current data in our study show that these devices are associated with an increased risk of complications being reported. Against the backdrop of potential harm to horse welfare associated with restrictive nosebands, this report may serve as a guide for future regulations and research. It helps improve our understanding of noseband preferences and their use in different disciplines.

## 1. Introduction

The performance of equine athletes depends on the optimal combination of good breeding, husbandry, rearing, training, preparation and horsemanship of their handlers, riders and trainers. The equipment used to train, restrain, steer and decelerate horses varies across disciplines, and has been designed for almost every part of a horse’s body. The greatest attention has been paid, however, to the horse’s head. Nosebands are one such piece of equipment used in many equestrian pursuits.

Nosebands originated in the military due to their potential use as a headcollar [1] and come in many traditional and contemporary designs. Today, they are commonly used to deter horses from opening their mouths and displacing their bit(s) [2]. Aesthetically, nosebands are said to “frame the horse’s face”, which is why some equestrians regard horses without them as “undressed” [2]. Nosebands may also be attached to the reins so that a rider can apply pressure to steer or decelerate their horse [3].

Tightening nosebands seems to result, at least in the short term, in the horse being more sensitive to rein tension [3]. The link between noseband tightness and rein tension may explain, in part, why nosebands may be applied so tightly as to be restrictive. The risk of over-tightening is amplified by the current lack of consistent international rules for monitoring noseband tightness. The International Society for Equitation Science (ISES) [4] have instituted a benchmark of a two-finger spacing, measured at the nasal midline (flush on the nose). Although this standard mirrors conventional industry practice, the benchmark is non-binding. The two-finger spacing is also recommended by a number of horse welfare organisations including the Royal Societies for the Prevention of Cruelty to Animals (RSPCA) in the UK and Australia, as well as The Horse Trust [5]. Despite this, the two-finger spacing benchmark is yet to be endorsed by the international equestrian and racing governing bodies [6].

Some national equestrian federations have recently specified that a minimum spacing is required under the noseband at the nasal midline. The Danish and Swiss Equestrian Federations, for example, stipulate a minimum distance of 15 mm should be achieved between the strap and the skin [7]. The Swedish Equestrian Federation proposes a distance of 20 mm. Although these new rules are a step in the right direction, they remain difficult to enforce and comply with until a standardised measuring tool is provided to stewards and riders [8]. In contrast, the British Dressage Rulebook follows the guidelines set by the FEI that state the tightness check must be performed with the steward’s index finger between the horse’s cheek and the noseband [9]. This location, however, is uninformative due to the much flatter and, in places, concave or hollow, shape of the horse’s face at the side [6,10].

Restrictive nosebands are primarily designed to keep the horse’s jaws closed and reduce behavioural signs of resistance, ranging from mouth-opening to tongue-lolling. These signs appear as the horse attempts to seek comfort, either by seizing the bit between its premolars or retracting its tongue to form an effective cushion to bit pressure. Both of these behaviours rely on the horse being able to open its mouth [11]. Limiting such responses is likely to increase discomfort from the bit(s) [12]. Thus, restrictive nosebands increase the rider’s control over the horse through a negative enforcement method [13]. Because restrictive nosebands can impose sustained pressures on soft tissues and bones, pressures that peak when the horse attempts to perform normal jaw movements, such as those involved in chewing, are thought to compromise welfare [14,15]. Crank nosebands that can be tightened by an in-built pulley system are the most restrictive design [16]. Concerns for horse welfare arising from restrictive tightening relate to the immediate effects of pressure on underlying tissues, pushing the horse’s cheek against the buccal surfaces (potentially causing abrasion or ulceration of the oral mucosa) and the suppression of normal behaviours, such as lip-licking, chewing, yawning and swallowing [10,14,17].

Horses are largely trained through negative reinforcement whereby an aversive stimulus is removed immediately upon the horse offering the desired response [18]. Relentless pressure defies any justification on the grounds of training. Restrictive nosebands can reduce or remove a horse’s ability to chew, swallow, yawn and lick its lips [17,19]. By definition, restrictive nosebands can violate the Five Freedoms of animal welfare [20], in that they are designed to suppress the expression of normal behaviour. Denying horses the ability to move their jaws in order to seek comfort when bit pressure is applied results in the horses’ inability to deal with aversive pressure from the bit(s) [10]. Indeed, the oral behaviours involved in responding to aversive pressures are precisely those resistances that are penalised in Dressage.

Within FEI-sanctioned horse sports, there are no consistent guidelines for noseband tightness. The Dressage guidelines for gear are arguably the most comprehensive, with detailed lists of the types of nosebands permitted, mandating double bridles at the elite level, and that “a noseband may never be so tightly fixed that it causes harm to the horse” [21]. However, until governing bodies articulate how nosebands may cause such harm, this guideline will be too vague to be enforced. Eventing, Show-jumping, Polo, Camp-drafting and Racing specify only what types of nosebands are permitted, but the acceptable tightness of nosebands for these disciplines is not explicitly stated [21,22].

It has been suggested that, through repeated use, restrictive nosebands can potentially lead to micro-fractures of the nasal bones [16] and bone remodelling [20]. However, to date, peer-reviewed evidence of this outcome is absent. That said, there is early circumstantial evidence to suggest that nasal bones may be at greater risk of deformation in Warmbloods using nosebands [23]. This is likely due to this type of horse being favoured in Eventing and Dressage [23], which are the two equestrian sports in which restrictive nosebands are commonly used. It has also been shown that excessive noseband tightness is associated with lip abrasions and/or blood at the commissures of the lips [24].

The putative reasons for noseband use are diverse and merit exploration across disciplines. This article reports on a survey designed to explore the types of nosebands that owners, riders and trainers use on horses in training and competition and their reasons for selecting these nosebands. The survey was developed to gain an understanding of approaches to noseband tightening and the incidence of complications.

## 2. Materials and Methods

### 2.1. Survey Instrument

Approval from the Human Research Ethics Committee of the University of Sydney was obtained prior to launching the current survey (Approval number: 2018/305). A draft questionnaire was piloted on volunteers (*n* = 20). Feedback prompted minor changes (such as to the tone and clarity of the questions) and led to the final questionnaire (a full transcript can be found following the URL in the Supplementary Materials section of this report).

The survey software (RedCap, Vanderbilt) allowed selective questions to be administered to participants within various horse disciplines. This questionnaire structure meant that the same survey could be disseminated across various interest groups. After asking respondents to specify their preferred equestrian pursuits, the survey focused on questions about the types of nosebands used, their purpose and details regarding their fitting. The survey ended with additional details about the respondents’ demographics, including their age, duration of involvement with horses and country of residence.

Respondents were first asked to describe their primary activity with horses, before stating how many horses they had, as well as what breed. To investigate who was using nosebands, respondents were asked whether they trained or raced any of their horses with nosebands. If they answered yes (i.e., *Always*, *Usually*, *Sometimes* or *Rarely*), participants were asked to select the type of noseband used from a list of illustrated options (Plain Cavesson noseband, Hanoverian noseband [with or without Flash], Drop noseband, Figure-of-eight/Grackle noseband, Micklem noseband, Sheepskin noseband or Other). Respondents were asked to specify whether or not their nosebands had a “cranking” system (that was illustrated in the questionnaire).

Respondents were then asked to answer three questions (responding on a scale of *Always*, *Usually*, *Sometimes*, *Rarely* and *Never*) outlining: whether they use tighter nosebands during competition than at home, whether they tighten their horse’s noseband immediately before or after competition, and whether or not their horse’s noseband is loosened for particular activities. Respondents were then asked to select up to 5 choices from 18 potential reasons for using nosebands: *to improve the rider’s/driver’s ability to decelerate the horse*; *to improve the rider’s/driver’s ability to steer the horse*; *to improve the rider’s/driver’s ability to put the horse on the bit or in a frame/outline*; *to prevent or reduce airway obstruction*; *to reduce airway noise*; *to improve performance in competition*; *to prevent the horse moving its tongue over the bit*; *to improve the horse’s acceptance of the bit/contact*; *to improve the appearance of the horse*; *to prevent the horse from opening its mouth*; *to prevent the horse crossing its jaws*; *to prevent the bit sliding through the horse’s mouth*; *to align with rules of the sport*; *the current noseband came with the bridle when I purchased it*; *my instructor/coach/friend told me I needed to use one*; *a veterinarian told me that I needed to use one*; *most people in my sport use them;* and *other*.

Respondents were also asked to comment on the noseband’s efficacy in achieving the stated purpose (selecting from *Extremely effective*, *Very effective*, *Effective*, *Somewhat effective* and *Not at all effective*), how long in minutes the noseband was usually left on and whether they noticed any undesirable physical or behavioural consequences from its use, for which the 13 options were: *hair loss in the area under the noseband*; *soreness in the area under the noseband*; *swelling of the area under the noseband*; *discoloration of the area under the noseband*; *bleeding from the mouth*; *lip injuries*; *reduced appetite*; *dropping food*; *behavioural signs of anxiety/distress*; *head shyness*; *difficulty bridling the horse* and *difficulty fastening the noseband;* and *other*. Next, respondents were asked whether they checked the tightness of the noseband and, if so, where on the horse’s head they conducted such checks. For this question, the available responses were as follows: *at the bridge of the nose; along the right or left cheek*; *under the chin;* and *other*. This question was followed by a question which prompted respondents to specify whether they employed a standardised taper gauge; selecting from: *Yes, I have used taper gauges and find them useful*; *Yes, I have used taper gauges and do not find them useful*; and *No, I have never use a taper gauge*.

### 2.2. Contact List Creation and Distribution

A database of breed and discipline associations, online magazines and individual Thoroughbred and Standardbred owners/trainers from both Australia and other English-speaking countries was compiled from web searches that included the Australian Yellow Pages website, Australian Racehorse Directory, broad governing associations (e.g., Equestrian Australia), and discipline or breed-specific associations. Respondents were encouraged to share the survey with their networks with the intention of further disseminating the survey. An article in the December issue of the Australian equestrian journal, Horses and People magazine, was commissioned to raise awareness among its subscribers. Additionally, various associations and individuals were contacted via email. Follow-up emails were circulated two months later. A Facebook page with details of the survey was posted to increase awareness of the survey through social media. This page and its contents were hosted on both the University of Sydney’s Veterinary Science and the University of Adelaide’s Equine Health and Performance Centre Facebook pages. Pamphlets were also distributed by hand during Equitana Melbourne in November 2018. The survey remained online for six months, from 28 August 2018 to 28 February 2019. A participation information statement directed participants to answer the questionnaire once only.

### 2.3. Data Analysis

Data were analysed using the “MASS” package [25] in the R statistical software environment [26]. Responses to the question “Do you train/race any of your horses while wearing nosebands?” were collected in the ordinal form of *Always*, *Usually*, *Sometimes*, *Rarely* and *Never*, and were analysed using ordinal logistic regressions, using the “POLR” function. Six potential explanatory factors, namely equine discipline, horse breed, country, rider age, rider gender and rider experience, were first assessed using univariate ordinal logistic regression. To balance model completeness with model parsimony, any explanatory variables having a likelihood ratio χ^2^ associated with a *p*-value less than 0.20 were considered for multivariable modelling. All potential explanatory variables qualified for multivariable modelling. All terms were placed in an additive model (the full model). Following this, the full model was trialled and the additive term with the lowest likelihood ratio χ^2^ (provided it was associated with a *p*-value less than 0.20) was dropped from the model, resulting in the terms “rider experience”, and when this process was repeated, “rider gender”, being dropped from the model.

The final model:

Noseband usage = equine discipline + horse breed + country + rider age(1)

did not explain significantly less deviance than the full six term model under the χ^2^ approximation (deviance difference = 28.425, df = 22, *p* = 0.16). In this model, the dependent variable was modelled as an ordinal response with five levels (*Always, Usually, Sometimes, Rarely* and *Never).* The independent variable equine disciple was modelled as a fixed factor with 12 levels: Western; Trail Riding; Showing/Hacking; Show-jumping; Racing, Pony Club/Working Equitation; Pleasure/Fun; Natural Horsemanship; Eventing; Endurance; Driving; Dressage; or Other. The dependent variable breed was modelled as a fixed factor with 18 levels: Andalusian, Appaloosa, Arabian, Australian Stock Horse, Cob, Connemara, CrossBreed, Icelandic, Irish Sport Horse, Morgan, Pony, Quarter Horse, Standardbred, Thoroughbred, Various, Warmblood, Welsh and Other. The independent variable Country was modelled as a fixed effect with 8 levels: Australia, Canada, Ireland, New Zealand, Sweden, United Kingdom, United States of America and Other. Finally, rider age was modelled as a fixed effect with 7 levels: 18–25, 25–35, 36–45, 46–55, 56–65, 66–75 and 76 years old or more. Coefficient estimates and standard errors from the model are reported in Section 3.1.1, Section 3.1.2 and Section 3.1.3.

The likelihood for nomination of reasons for noseband use and for observing a complication from noseband use were evaluated using a binary logistic regression model using the “GLM” function of the “stats” package. For analysis, reasons for noseband use were grouped as “Anatomical”, “Consequential” and “Passive”. The Anatomical reasons were to: *prevent the horse from moving its tongue over the bit*; *opening its mouth*; *crossing its jaws*; and *prevent the bit sliding through the horse’s mouth*. The Consequential reasons were to: *improve the rider’s/driver’s ability to decelerate the horse*; *improve the rider’s/driver’s ability to steer the horse*; *improve the rider’s/driver’s ability to put the horse on the bit or in a frame/outline*; *prevent or reduce airway obstruction*; *to prevent or reduce airway noise*; *to improve performance in competition*; *improve the horse’s acceptance of the bit/contact;* and *improve the appearance of the horse*. The Passive reasons were: *to align with the rules of the sport*; *the current noseband came with the bridle when I* (the respondent) *purchased it*; *my instructor/coach/friend told me I needed to use one*; *a veterinarian told me that I needed to use one* and *most people in my sport use them;* and *other*.

The binary logistic regression models used were:

reason of type i = noseband usage + equine discipline,(2)

Complication observed = noseband usage + equine disciple + noseband type(3)

where “i” indicates a positive nomination by that respondent of any reason classed as Anatomical. Here, frequency of noseband usage (the dependent ordinal variable in the previous model) was fit as an unordered fixed effect with five levels: *Always*, *Usually*, *Sometimes*, *Rarely* and *Never*. The independent variable of equine disciple corresponded to all disciplines shown above with at least 40 respondents (Dressage, Pleasure/Fun, Trail Riding, Show-jumping, Eventing, Pony Club/Working Equitation, Natural Horsemanship, Racing, Showing/Hacking, and the remaining disciples pooled with Other, creating a 10-level factor). Noseband Types were: Plain Cavesson, Drop Noseband, Figure-of-Eight/Grackle noseband, Hanoverian Noseband, Micklem Noseband, Sheepskin noseband or other. Odds ratios (OR) and confidence intervals (CI) derived from the coefficients and standard errors are shown in Section 3.4 and below. The model was then fitted separately for Consequential and again for Passive reasons.

The proportional log odds assumption was assessed by graphically inspecting predictions of binary logistic regressions across each of the ordinal logistic regression cut points. Diagnostic surrogate-based residual plots, created by the “sure” package in R [27], were inspected.

Due to the different number of reasons within each category (four for Anatomical, seven for Consequential and five for Passive), a weighted value, calculated by taking an average of each category, was used when comparing categories. Data from all disciplines with at least 40 respondents (Dressage, Pleasure/Fun, Trail riding/Pleasure riding, Show-jumping, Eventing, Pony Club/Working Equitation and Natural Horsemanship) were examined using logistic regression, and were examined further through descriptive analyses physical and behavioural complications.

## 3. Results

### 3.1. Responses

A total of 3236 respondents interacted with the questionnaire, with some completing the questionnaire to varying degrees. To simplify statistical analysis, data which were deemed incomplete within critical criteria (noseband usage, equine discipline, horse breed, age and country) were disregarded from testing, with 196 respondents fitting into this category. The testable sample for this study was therefore 3040 respondents, with all future sections to be analysed in respect to this sample, with 2332 respondents reporting some level of noseband use.

#### 3.1.1. Respondent Demographics

The distribution of the 3040 respondents according to country was: Australia (35.1%, *n* = 1068), Sweden (23.3%, *n* = 709), New Zealand (12.7%, *n* = 387), United Kingdom (10.9%, *n* = 332), United States of America (4.3%, *n* = 132), Canada (3.1%, *n* = 93), Ireland (0.6%, *n* = 18), and Other (9.9%, *n* = 301). The distribution of the age of respondents was: 18–25 (16.6%, *n* = 505), 26–35 (21.7%, *n* = 659), 36–45 (21.1%, *n* = 642), 46–55 (23.0%, *n* = 700), 56–65 (14.3%, *n* = 435), 66–75 (2.9%, *n* = 89) and 76–85 (0.3%, *n* = 10). The distribution of sex of our respondents was mostly female (96.2%, *n* = 2925), followed by male (3.3%, *n* = 99), with 0.6% (*n* = 16) electing not to answer this question.

#### 3.1.2. Noseband Use

For most countries of origin (United States of America, Sweden, New Zealand, Ireland, Canada and Other), there were no differences in frequency of noseband usage. In contrast, UK respondents (OR = 0.55; 0.43–0.71) were more likely to use nosebands than the study average, while Australian respondents (OR = 1.41; 1.18–1.68) were less likely to use nosebands than the study average.

Older riders were more likely to report not using nosebands than those in the 18–25-year-old age group. With an increase in age, the likelihood of using nosebands declined. Respondents involved in Natural Horsemanship (OR = 5.47; 3.68–8.14) and Western (OR = 3.26; 1.84–5.77) disciplines were significantly less likely to use nosebands than the other disciplines (Table 1).

The distribution of respondents’ noseband use was as follows: 39.6% (*n* = 1203) *always* used nosebands, 18.7% (*n* = 567) *usually* used nosebands, 11.4% (*n* = 346) *sometimes* used nosebands, 7.1% (*n* = 216) *rarely* used nosebands and 23.3% (*n* = 708) *never* used nosebands. If respondents reported that they did not always use nosebands (60.4%, *n* = 1837), they were asked to clarify the reasons why. When prompted to clarify their non-use of nosebands, most of the respondents who did not always use nosebands (50.4%, *n* = 868) on their horses stated that there was no need, with the next highest selection being that respondents wanted their horses to be able to open their mouths (39.3%, *n* = 677). Only 0.5% (*n* = 9) stated that the rules of their sport prohibit the use of nosebands, with 9.8% (*n* = 168) respondents stating that they did not use nosebands for a reason not provided by the survey. Non-users of nosebands or those who selected *Never* (*n* = 708) according to discipline were ranked as follows: Trail riding/Pleasure riding (27.0%, *n* = 191), Pleasure/Fun (22%, *n* = 156), Other (17.9%, *n* = 127), Dressage (12.7%, *n* = 90), Natural Horsemanship (10.2%, *n* = 72), Pony Club/Working Equitation (3.8%, *n* = 27), Showing/Hacking (1.8%, *n* = 14), Eventing (1.4%, *n* = 10) and Racing (1.1%, *n* = 8).

Of those respondents who used nosebands (*n* = 2332), 46.6% (*n* = 1087) used a Plain Cavesson, 24.8% (*n* = 579) used a Hanoverian noseband, 13.8% (*n* = 322) used a Micklem noseband, 7.1% (*n* = 165) used a Drop noseband, 7.1% (*n* = 72) used a Figure-of-Eight/Grackle noseband and 0.4% (*n* = 10) used a Sheepskin noseband, with only 3.5% (*n* = 81) using another type of noseband not listed, such as a bitless bridle.

Explanatory variables associated with significantly lower noseband usage, i.e., protective variables, are shown in Table 1. Explanatory variables associated with significantly higher noseband usage, i.e., risk factors, are shown in Table 2.

#### 3.1.3. Breed Differences

Even with discipline included in the model, various breeds showed significantly more or less likelihood of noseband use than the study average. Stock and Western breeds, the Australian Stock Horse (OR = 3.14; 2.07–4.75) and the Quarter Horse (OR = 2.88; 2.00–4.15) were significantly less likely to wear nosebands. In contrast, many breeds of pony, Thoroughbreds and sports breeds (such as Warmbloods or Icelandic), were more likely to wear nosebands.

#### 3.1.4. The Main Reasons for Noseband Use Offered by Respondents

The respondents who used nosebands had the option to select up to five reasons for why they used a noseband. Within this section, 5092 individual responses recorded reasons for noseband use. This section produced a high response rate with the mean number of responses per participant being 1.7, and the modal number offered by respondents being 1. Only 36 respondents who used a noseband did not offer a single reason for use.

The distribution of these reasons appears in Table 3, with the most common reasons given being Passive reasons (32.9%, *n* = 1673). Within this group, the most common reasons were *to align with the rules of the sport* (30.2%, *n* = 705) and *the current noseband came with the bridle when purchased* (24.7%, *n* = 576). Within the Anatomical group, the reason *to prevent the horse from moving its tongue over the bit* (20.8%, *n* = 485) was most common while, among the Consequential reasons, *to improve the appearance of the horse* (20.4%, *n* = 476) was the most common response.

Plain Cavessons were more likely to be used *to align with the rules of the sport* (35.2%, *n* = 383) than others, such as the Hanoverian (28.8%, *n* = 167) or Micklem (27.0%, *n* = 87).

For *the bridle came with the current noseband*, Plain Cavesson (28.7%, *n* = 312), Micklem (35.1%, *n* = 113) and Hanoverian nosebands (19.2%, *n* = 111) comprised the majority of the responses.

Other responses were offered by nearly one in nine respondents (7.0% of overall responses). Some respondents also stated that their use of nosebands was for stabilisation (*n* = 24, of which 11 specified stabilisation of the bit, six specified stabilisation of the bridle, seven used the noseband to attach a fly guard/nose net, three for the stabilisation of blinkers, and one to attach a lugging pole). These were pooled into the *it came with the bridle* option to simplify analysis.

### 3.2. Distribution of Noseband Types across Disciplines

From the list of seven noseband choices offered to participants, the reported use of each design for disciplines with 40 or more responses is shown in Table 4 (16 respondents who specified their discipline elected not to select their noseband type and thus were not included). Plain Cavesson nosebands were the most commonly selected across all disciplines. More than 50% of respondents in the less popular disciplines (those disciplines with relatively low numbers of respondents), such as hacking and Natural Horsemanship, reported using nosebands.

Among the more popular disciplines, such as Dressage, Eventing and Show-jumping, Hanoverian nosebands were the most common, with 31.9% in Dressage, 34.8% in Show-jumping and 33.0% in Eventing. Grackle nosebands were relatively common in Racing (compared to the rest of the noseband types), with 30.0% of respondents in this discipline using them.

### 3.3. How Respondents Reported Noseband Tightness and Whether They Used a Crank Tightening System

For respondents who answered the question “Do you check the tightness of nosebands?” (*n* = 2312), most (96.3%, *n* = 2226) stated that they checked their nosebands *always* or *usually*; 1.8% (*n* = 41) of respondents reported that they only *sometimes* checked the tightness of nosebands; 0.9% (*n* = 20) stated that they *rarely* check; and only 1.1% (*n* = 25) reported that they *never* checked. Respondents (*n* = 2307) also reported on whether they checked for noseband tightness in the space between the noseband and the skin. This category produced a similar distribution of responses to the preceding question, with 96.2% (*n* = 2219) stating that they *always* or *usually* checked in this way, with 1.9% (*n* = 43) reporting that they only *sometimes* checked in this way, 1.0% (*n* = 22) reporting that they *rarely* checked in this way and 1.0% (*n* = 23) stating that they *never* checked in this way.

#### 3.3.1. Where on the Horse’s Head Did Respondents Check for Tightness?

Site of checking was reported by 2295 respondents. Most of these respondents (62.1%, *n* = 1426) indicated that they check tightness at the bridge of the nose, 10.4% (*n* = 238) at either cheek, 21.4% (*n* = 490) under the chin and 6.1% (*n* = 141) at other places.

#### 3.3.2. Did Respondents Use a Crank Tightening System?

Of the 2332 respondents who used nosebands, 28.9% (*n* = 665) reported using a crank tightening system on their nosebands, but 2.2% (*n* = 50) indicated they did not know. The respondents within each discipline who answered whether they use a crank tightening system in the affirmative were ranked as follows: Dressage (39.2%, *n* = 310), Natural Horsemanship (33.3%, *n* = 12), Showing/Hacking (29.4%, *n* = 20), Eventing (30.1%, *n* = 63), Show-jumping (23.8%, *n* = 62), Trail riding/Pleasure riding (23.7%, *n* = 44), Racing (24.0%, *n* = 12), Pony Club/Working Equitation (23.3%, *n* = 37), Pleasure/Fun (20.6%, *n* = 74) and Other (16.7%, *n* = 31).

#### 3.3.3. Tightness of Nosebands at Home Compared to Competition

In response to being asked whether they used tighter nosebands at competition than at home, most participants more frequently reported using the same noseband tightness at home, when training the horse and during competition, than tightening it for competition (Table 5). Due to the logic of the questionnaire software, respondents who did use nosebands were offered these questions regardless of whether they specified the type of noseband use, which resulted in nine respondents not answering this question.

#### 3.3.4. Taper Gauge Use by Respondents

When prompted with a visual of a taper gauge, most respondents who used nosebands and answered this question, 95.9% (*n* = 2222), did not know what it was. Only 3.4% (*n* = 79) noted that they have used (or currently use) a taper gauge and find it to be helpful, with the remaining 0.7% (*n* = 17) noting that they had used a taper gauge in the past and did not find it useful.

### 3.4. Links between Discipline and the Reasons for Noseband Use

The relationships between discipline and the reason for noseband use are presented in Table 6. Respondents who trained racehorses (OR = 2.62; 1.54–4.46) and show-jumpers (OR = 1.37; 1.06–1.76) were more likely to give an Anatomical reason for noseband use than the average, whereas respondents involved with Natural Horsemanship (OR = 0.49; 0.25–0.99) and Pleasure/Fun (OR = 0.64; 0.50–0.80) were less likely to give an Anatomical reason. Additionally, respondents who trained racehorses (OR = 2.93; 1.68–5.13) were more likely to give a Consequential reason for noseband use than the average, whereas Dressage riders (OR = 0.83; 0.70–0.99) and those involved with Pleasure/Fun (OR = 0.59; 0.47–0.73) were less likely to give a Consequential reason. Dressage riders (OR = 1.41; 1.18–1.68) and Eventing riders (OR = 1.48; 1.13–1.90) were more likely to offer a Passive reason for noseband use than the average, whereas those who trained racehorses (OR = 0.35; 0.20–0.62) were less likely to give a Passive reason.

### 3.5. What Is the Relationship between the Type of Noseband Used and the Reasons for Use?

The relationships between types of nosebands used and the reporting of at least one of each type of reason are shown in Table 7, noting that, as in Section 3.2, 16 respondents did not specify the type of noseband they use. The highest percentage of Passive reasons for noseband use came from respondents who were using Micklem (59.3%), Hanoverian (50.8%) or Plain Cavesson nosebands (59.8%). Apart from those using less commonly reported nosebands (such as Sheepskin nosebands, *n* = 10), at least half of respondents using each noseband type reported at least one Anatomical reason for their choice of noseband. Respondents using Drop nosebands were the most likely to cite an Anatomical reason for doing so (70.9%). At least half of participants using Hanoverian, Figure-of-Eight/Grackle and Drop nosebands all reported doing so for Consequential reasons. The distribution of Anatomical, Consequential and Passive reasons is presented in Table 7.

### 3.6. Effectiveness of Nosebands

**Figure 1:**
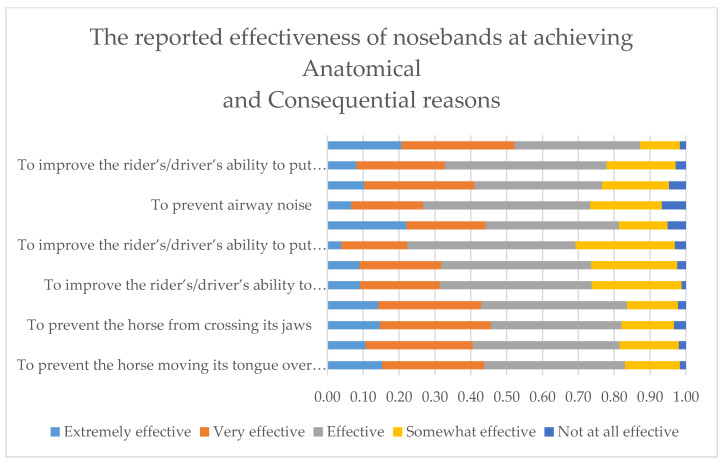
The reported relative effectiveness of nosebands at fulfilling the Anatomical (first four listed reasons) and Consequential (the next eight) reasons for their use. Anatomical reasons for noseband use: *To prevent the horse moving its tongue over the bit*, *To prevent the horse opening its mouth*, *To prevent the horse crossing its jaws* and *To prevent the bit sliding through the horse’s mouth*. Consequential reasons for noseband use: *To improve the riders/drivers ability to decelerate the horse*, *To improve the riders/drivers ability to steer the horse*, *To improve the riders/drivers ability to put the horse on the bit or in a frame/outline*, *To prevent or reduce airway obstruction*, *To prevent or reduce airway noise*, *To improve performance in competition*, *To improve the horse’s acceptance of the bit/contact* and *To improve the appearance of the horse*. Effectiveness was reported 3040 times by respondents who used nosebands (*n* = 2332).

The effectiveness of nosebands in delivering specific consequences or Anatomical outcomes is shown in Figure 1.

### 3.7. Nosebands and Complications

Of the 2332 respondents who used a noseband, 18.6% (*n* = 434) reported having seen at least one behavioural or physical complication. Among these, the most commonly reported complication was hair loss under the noseband (31.3%, *n* = 173). The representation of these and other complications are shown in Table 8.

There was no association between discipline and reports of adverse effects. Respondents who currently use a Micklem bridle were at higher odds than average of reporting they had seen a complication for noseband use at some time than users of Plain Cavesson nosebands (OR = 1.48; CI 1.09–1.99). Users of all other noseband types were not associated with having encountered a noseband complication.

#### 3.7.1. Are Complications Related to Noseband Use?

Respondents who reported *rarely* using a noseband were at higher odds than those who reported *always* using a noseband of stating they had seen a complication from noseband use at some time (OR = 1.53; 1.10–2.13).

#### 3.7.2. Are Crank Nosebands More Likely to Result in a Complication?

Out of 1589 respondents who did not use a crank-like device on their nosebands, 28.6% (*n* = 613) reported at least one complication. Out of 665 respondents who did use a crank-like device on their nosebands, 41.7% (*n* = 277) reported at least one complication.

## 4. Discussion

With 3040 respondents, this study is one of the most comprehensive to date on the subject of noseband use across a variety of equestrian disciplines and racing. It shows that reasons for noseband use vary considerably and that the reported effectiveness of nosebands depends on their design and the context in which they are being used. Among the respondents, 2332 used nosebands. Those who did not were most likely to be engaged in Natural Horsemanship, Western riding and Endurance. This may be due, in part, to these sports either not requiring noseband use or not penalising those displays of discomfort such as mouth-opening which nosebands can reduce. Of the 1837 participants who answered that they did not always use nosebands, most (50.4%, *n* = 868) stated that there was no need. Many of these respondents (39.3%, *n* = 677) stated that they wanted their horses to be able to open their mouths, with only a small number (0.5%, *n* = 9) stating that nosebands were not allowed in their discipline.

Of the six different noseband types (with *other* types as a seventh option), three contributed to 85.2% (*n* = 1988) of responses. Plain Cavesson nosebands were the most popular in all disciplines except Show-jumping. In addition, they were significantly associated with disciplines for which there were fewer respondents, such as Camp-drafting and Polo. The predominance of Passive reasons given for Plain Cavesson noseband use (59.8%, *n* = 650) indicates that they are generally perceived as the “standard” noseband. The relatively low number of Anatomical reasons given (23.6%, *n* = 257) for Plain Cavesson noseband use further supports the view that there is often no specific motive for their use.

Hanoverian nosebands were the most popular noseband type in Dressage, Show-jumping, Eventing and Hunting. These nosebands are effectively a variation of the Plain Cavesson which can mount a second strap that is adjusted below the bit. Either one or both the straps in such an assembly can be tightened enough to restrict the horse’s mouth movements [14]. In the current study, Hanoverian nosebands were mainly used for Anatomical reasons (62.0%, *n* = 359), consistent with an intention to restrict the horse’s oral behaviours.

In the current study, Grackle (or Figure-of-eight) nosebands were used almost exclusively for Racing. Grackle nosebands differ from Plain Cavesson chiefly because they disperse pressure more evenly across the front of the head and the upper strap can be positioned more proximally than the straps of other noseband designs. With this design, they are believed to interfere less with movement and expansion of the nostrils during exercise, allowing greater air intake [28]. They may also aid in the prevention of certain respiratory issues (such as dorsal displacement of the soft palate (DDSP)), by keeping the mouth closed and thus preventing air from entering the oral cavity and contributing to palatal instability [28,29,30]. A study of 750 Eventing, Dressage and performance hunter horses found that Grackle nosebands were applied especially tightly, with a median spacing between the strap and skin of 0.5 fingers, and a mean spacing of 0.7 fingers at the nasal midline [6].

Over one quarter of respondents (28.9%, *n* = 665) reported using a crank tightening system. Crank nosebands are particularly problematic from an equine welfare perspective because they can clamp the jaws of the horse shut with considerable pressure. This pressure peaks as horses try to chew [14]. Additionally, the forces applied to the underlying soft tissue and nerves merit concern, given that nosebands compress soft tissue areas directly below them [6]. Importantly, respondents who used a crank-like device were more likely than their non-crank-using counterparts to report one or more complications (41.7%, *n* = 277 compared with 28.6%, *n* = 613).

The most common Anatomical reason given for using nosebands was *to prevent the horse from moving the tongue over the bit* (20.8%, *n* = 485). The bit is an instrumental part of the bridle that allows the rider to apply pressure to the horse’s tongue and diastema to facilitate steering and deceleration [31]. If a horse can move its tongue over the bit, then one of the primary points of contact/pressure from the rider is transferred to the soft tissues covering the mandible. In this position, the bit may cease to function properly which may increase the risk to the rider if the horse is unreceptive [32]. Without proper bit function, the rider risks losing control of the horse. This is of critical importance especially in high-speed activities such as Show-jumping, Eventing and Racing, where injury rates are higher than in other sports [33]. Nevertheless, overall, fewer than 8% of respondents stated they primarily used nosebands for steering (7.2%, *n* = 168) and deceleration (7.5%, *n* = 175). This result may be of interest to horse-sport regulators who, when attempting to enforce loosening of nosebands for horse welfare reasons, may face resistance on the grounds that tight nosebands are needed for safety. If riders are not employing nosebands for steering and deceleration purposes (i.e., for safety reasons), enforcing the traditional two-finger fit should be adopted on welfare grounds.

Preventing the horse from opening its mouth (17.8%, *n* = 413) or the bit from sliding through its mouth (17.7%, *n* = 412) were almost as common a response as preventing the horse from moving the tongue over the bit (20.8%, *n* = 485), which confirms that the bit is of central importance to those who train and ride horses. That said, it is less clear how those who ride without nosebands remain safe while still achieving these bit-related objectives.

The most frequent Consequential reason for noseband use was to improve the appearance of horses. Plain Cavessons were far more likely to be used for this purpose (29.9%, *n* = 328) compared with other nosebands, such as the Grackle (11.1%, *n* = 8), which was more likely to be chosen by Racing respondents to improve the steering and deceleration of the horse.

The current findings revealed that Dressage and Eventing respondents most frequently offered Passive reasons for noseband use (60.1% and 59.3%, respectively). This may run counter to the expectation that participants in these disciplines would have different reasons to use nosebands. Specifically, some might expect Dressage riders to use nosebands to give the impression of their horses showing “submission” and “acceptance of the bit”, whereas Eventing riders maybe seeking to increase control of the horse.

Our results showed only 4.1% (*n* = 96) of respondents were familiar with the ISES taper gauge. It would be useful to know the factors that influence this device’s take-up in sports, especially in light of our findings that respondents did not check for tightness in any measurable/consistent manner. It is worth noting that the Danish Equestrian Federation has introduced a 15 mm gauge to be used to check noseband tightness at the bridge of the nose of horses competing in all sports [34]. Furthermore, Dressage New Zealand has also taken steps to limit noseband tightness in competition, where a minimum of one finger must be able to fit under the noseband at the bridge of the nose at all times [35]. It is encouraging that some federations are taking the initiative in demanding that these spacing guidelines be stated and enforced.

Our results align roughly with a previous study in which 9.2% of horses were observed with abrasions and/or blood visible at the commissures of the lips, although only 4.6% (*n* = 20) of the current respondents reported having observed this outcome [23]. The same previous study further reported that tighter nosebands were associated with a higher frequency of commissural lesions. Complete absence of the noseband (which is permitted in some disciplines) did not abolish ulcerations at the labial commissures but was associated with increased risk compared with a loosely adjusted noseband. The authors hypothesised that, without a noseband, the horse can resist bit pressure by opening the mouth, an action that facilitates abrading tissues between the bit rings/cheeks and the teeth. The authors concluded that, to be beneficial, the noseband should not be adjusted too tightly.

Complications relating to noseband use could impact a respondent’s willingness to continue using the same design of noseband. This is supported by the current finding that those who *Rarely* use nosebands were far more likely to report a complication due to noseband use, compared with those who *Always* use nosebands.

Nosebands can press the mucous membranes against the sharp buccal margins of the premolars, thus increasing the risk of abrasion and ulceration within the oral cavity. Interestingly, respondents who used Micklem nosebands were more likely than the average to report physical complications with noseband use. However, Micklem nosebands were designed to avoid compressing the sensitive areas of a horse’s head [36]. A possible explanation for the current finding could be that respondents using Micklem nosebands have elected to use this design because they have encountered problems with other designs, a prospect that warrants further investigation.

Overall, 30.2% (*n* = 705) of respondents stated that they use nosebands because the rules of the sport require them to. In the disciplines of Eventing, Show-jumping and Dressage, 95.3% (*n* = 488) of respondents stated this as their main reason, although it is only in Dressage and the Dressage phase of Eventing where nosebands are obligatory [21]. Some respondents (24.7%, *n* = 579) stated that their bridle came with their specified noseband. In combination, these findings could reflect a lack of awareness of the potentially deleterious consequences of noseband use. Furthermore, if nosebands are mandated in certain disciplines and may have deleterious physical and behavioural impacts on horses, it follows that sports governing bodies should establish evidence-based and clear guidelines regarding their use and adjustment. Rules 2.1.5, 2.2, 2.2.1 and Annex 16 in the FEI rules for Dressage stipulate that “a noseband should never be so tightly fixed that it causes harm to the horse” [21] but fail to articulate either how it causes harm or how tightness should be measured. However, the FEI Steward guidelines (2019) for monitoring tightness and adjustment fail to meet the evidence-based recommendations of establishing a minimum spacing at the nasal plane and monitoring compliance using an objective gauge, despite studies such as Doherty et al. (2016) suggesting that nosebands are generally overtightened [6,37].

The prevalence of Passive reasons for noseband use among our respondents is somewhat surprising, given the suggested competitive advantages of restrictive nosebands [17]. If gaining a competitive advantage is the main motivation for noseband usage, one would expect a higher percentage of Consequential reasons should have been observed in the current results. It is worth noting that only 12.5% of respondents (*n* = 289) admitted to tightening their horses’ nosebands immediately before competing. It may be that most riders do not adopt this practice because they are satisfied that the current level of tightness during warm-up and training is ideal for competition, they are unaware that escalating the tightness between warming-up and competing may increase sensitivity to the bit(s) or the level of tightness they use for warm-up and training is already maximal.

It is important to acknowledge some limitations with the current study. Due to this survey being anonymous and online, it demanded a level of trust in respondents to answer truthfully and to the best of their ability. Respondents were encouraged to read through a user agreement which described the nature of the survey and asked for their consent to participate in the study. Beyond that, this type of survey may reflect some residual bias, with respondents potentially being unwilling to comment honestly on the tightness of their horses’ nosebands, particularly when there has been considerable commentary in the social media about noseband tightness and the noseband taper gauge. Furthermore, the authors acknowledge that this should not be considered a definitive global survey, rather it is an opportunity to explore relationships among management variables and reported outcomes. Reported outcomes may be affected by cognitive dissonance. For example, respondents may wish to believe (and therefore report) that an item of gear achieves what they had hoped, even if it fails to so. Additionally, the authors acknowledge the limitations of internet surveys and the potential for non-responder bias. The authors accept that the distinctions between the three groups of reasons (Anatomical, Consequential and Passive) are not absolute and that some may overlap with one another. However, respondents were not confined to selecting only one reason for using nosebands and the labels we have used should not be considered definitive. Additionally, the logic pathways could be optimised for future studies.

The survey asked: *Do you check the tightness of nosebands?* To assess frequency of noseband checking, e.g., after bridles have been deconstructed for cleaning and then reassembled for use, it should have asked how often respondents check the tightness of nosebands. The current study failed to establish how tightly nosebands were being fitted. It is important to also note that we cannot make claims of direct causality from the current cross-sectional data. Furthermore, we acknowledge that oral discomfort and soft tissue complications to the head may have myriad causes beyond tight nosebands. The authors acknowledge that the questionnaire should have asked participants who noted consequences to specify which noseband they were using at the time of these consequences. In this vein, future studies should also attempt to isolate nosebands as a definitive cause of the complications noted by respondents. The nature of cross-sectional surveys such as the current one means that definitive causal effects cannot be divined. The low uptake of the ISES taper gauge in the current sample of respondents contributed to the absence of useful estimates of current practice in noseband checking. Further studies should explore the merits of asking horse-keepers to use a standard gauge when estimating the level of noseband tightness that they regard as acceptable. Further research should also seek to clarify why so many respondents gave Passive reasons for noseband use in equestrian activity. Finally, future researchers could benefit from a focused study of those engaged in Eventing, Dressage and Show-jumping as disciplines in where restrictive nosebands could be beneficial to performance, to determine how tight trainers apply nosebands to their horses, and, indeed, whether their noseband use is effectively “restrictive”. Additionally, further research should ascertain whether there are significant differences between countries. Finally, due to the complicated real-world relationships among breed, equipment, discipline and husbandry, further research is needed to tease out these effects.

## 5. Conclusions

This study reveals some of the reasons why riders and trainers use nosebands. Plain Cavesson nosebands were the most widely used in most disciplines, but the current findings provide evidence of preference for some designs over others in certain disciplines, such as a preference for Hanoverian nosebands in Eventing, Show-jumping and Dressage, as well as Grackle nosebands in Racing. The current prevalence of Passive reasons reported for noseband use suggests that, in general, nosebands are not chiefly used for a competitive advantage. While there was an even distribution of Anatomical, Consequential and Passive reasons for noseband use, within each discipline there was variation. The current results confirm that nosebands are often used to prevent the horse’s tongue from moving over the bit and to improve the appearance of the horse. The study also revealed some risks associated with noseband use, with almost a fifth of respondents reporting harmful consequences. Crank systems were reported in 28.9% of respondents’ nosebands. This is of concern because these devices can be excessively tightened, minimising jaw and tongue movement and may compromise horse welfare. This concern is borne out by the increased rate of reporting complications by crank-using respondents compared with their non-crank-using counterparts. Finally, this survey provides evidence that although most respondents check for tightness at the bridge of the nose, many still check for tightness in uninformative locations.

## Figures and Tables

**Table 1:** Explanatory variables (equestrian disciplines, horse breeds, country of origin and respondent ages) associated with significantly increased odds of less noseband use compared with riders 18–25 for age, or the study mean for other types of variable.

Explanatory Variables	Odds Ratio	95% Confidence Interval of Odds Ratio	
		2.5%	97.5%
Disciplines			
Natural Horsemanship (*n* = 108)	5.47	3.68	8.14
Western (*n* = 49)	3.26	1.84	5.77
Endurance (*n* = 60)	2.61	1.57	4.32
Trail Riding (*n* = 385)	1.87	1.51	2.31
Pleasure/Fun (*n* = 518)	1.22	1.01	1.47
Breeds			
Australian Stock Horse (*n* = 83)	3.14	2.07	4.75
Quarter Horse (*n* = 141)	2.88	2.00	4.15
Andalusian (*n* = 108)	1.52	1.06	2.17
Arabian (*n* = 161)	1.48	1.07	2.04
Country			
Australia (*n* = 1068)	1.41	1.18	1.68
Respondent age range (years)			
25–35 (*n* = 659)	1.41	1.12	1.78
36–45 (*n* = 642)	1.62	1.28	2.03
46–55 (*n* = 700)	2.15	1.70	2.71
56–65 (*n* = 435)	2.42	1.86	3.15
66–75 (*n* = 89)	3.11	1.98	4.89

**Table 2:** Explanatory variables (equestrian disciplines, horse breeds and country of origin) associated with significantly lower odds of less noseband use compared with the study mean.

Explanatory Variables	Odds Ratio	95% Confidence Interval of Odds Ratio	
		2.5%	97.5%
Disciplines			
Show-jumping (*n* = 277)	0.30	0.23	0.39
Dressage (*n* = 890)	0.35	0.29	0.41
Eventing (*n* = 219)	0.39	0.30	0.50
Showing/Hacking (*n* = 82)	0.48	0.33	0.71
Pony Club/Working Equitation (*n* = 186)	0.51	0.39	0.66
Breeds			
Connemara (*n* = 53)	0.51	0.31	0.84
Pony (*n* = 75)	0.52	0.34	0.79
Warmblood (*n* = 770)	0.55	0.46	0.66
Thoroughbred (*n* = 496)	0.67	0.55	0.82
Welsh (*n* = 87)	0.63	0.42	0.93
Icelandic (*n* = 100)	0.55	0.38	0.80
Country			
United Kingdom (*n* = 332)	0.55	0.43	0.70

**Table 3:** The distribution of reasons (*n* = 5092) that respondents (*n* = 2332) offered for use of nosebands. The percentage of all respondents using nosebands who selected a particular value are presented in the third column. The percentage of all reasons given by respondents (independent of the respondent) are presented in the fourth column.

Reasons Given for Noseband Use	*n*	% of Respondents	% of Reasons
**Anatomical reasons**			
To prevent the horse moving its tongue over the bit	485	20.8	9.5
To prevent the horse opening its mouth	412	17.7	8.1
To prevent the horse crossing its jaws	184	7.9	3.6
To prevent the bit sliding through the horse’s mouth	420	18.0	8.2
Subtotal	1501		29.5
**Consequential reasons**			
To improve the rider’s/driver’s ability to decelerate the horse	175	7.5	3.4
To improve the rider’s/driver’s ability to steer the horse	168	7.2	3.3
To improve the rider’s/driver’s ability to put the horse on the bit or in a frame/outline	133	5.7	2.6
To prevent or reduce airway obstruction	60	2.6	1.2
To prevent or reduce airway noise	15	0.6	0.3
To improve performance in competition	109	4.7	2.1
To improve the horse’s acceptance of the bit/contact	424	18.2	8.3
To improve the appearance of the horse	476	20.4	9.3
Subtotal	1560		30.6
**Passive reasons**			
To align with the rules of the sport	705	30.2	13.8
The current noseband came with the bridle when I purchased it	576	24.7	11.3
My instructor/coach/friend told me I needed to use one	118	5.1	2.3
A veterinarian told me that I needed to use one	11	0.5	0.2
Most people in my sport use them	263	11.3	5.2
Subtotal	1673		32.9
**Other**	358	15.4	7.0
Total	5092		100.0

**Table 4:** Distribution of each type of noseband across each discipline with at least 40 respondents (total *n* = 2316). The numbers in each cell represent the overall distribution of the type of noseband (columns) within each respective discipline (rows). “Other” categories include Driving, Endurance, Western, Polo/Polocrosse, Hunting, Mounted Games/Horseball and Mustering/Farm Work.

Discipline	Plain Cavesson	Drop	Figure-of-Eight/Grackle	Hanoverian	Micklem	Sheepskin	Other
Dressage (*n* = 800)	43.1% (*n* = 343)	8.4% (*n* = 67)	0.8% (*n* = 6)	32.0% (*n* = 255)	13.2% (*n* = 105)	0.3% (*n* = 2)	2.3% (*n* = 18)
Show-jumping (*n* = 264)	32.7% (*n* = 86)	6.1% (*n* = 16)	6.8% (*n* = 18)	35.0% (*n* = 92)	18.6% (*n* = 49)	0.0% (*n* = 0)	0.8% (*n* = 2)
Eventing (*n* = 209)	39.2% (*n* = 82)	2.4% (*n* = 5)	7.2% (*n* = 15)	33.0% (*n* = 69)	17.2% (*n* = 36)	0.0% (*n* = 0)	1.0% (*n* = 2)
Racing (*n* = 50)	46.0% (*n* = 23)	4.0% (*n* = 2)	30.0% (*n* = 15)	12.0% (*n* = 6)	2.0% (*n* = 1)	2.0% (*n* = 1)	4.0% (*n* = 2)
Natural Horsemanship (*n* = 40)	63.9% (*n* = 23)	0.0% (*n* = 0)	0.0% (*n* = 0)	16.7% (*n* = 6)	5.6% (*n* = 2)	0.0% (*n* = 0)	13.9% (*n* = 5)
Pleasure/Fun (*n* = 360)	50.6% (*n* = 182)	9.2% (*n* = 33)	2.2% (*n* = 8)	14.2% (*n* = 51)	16.4% (*n* = 59)	0.8% (*n* = 3)	6.7% (*n* = 24)
Trail Riding (*n* = 191)	55.6% (*n* = 104)	10.2% (*n* = 19)	0.5% (*n* = 1)	14.4% (*n* = 27)	12.3% (*n* = 23)	0.5% (*n* = 1)	6.4% (*n* = 12)
Pony Club/Working Equitation (*n* = 161)	54.7% (*n* = 87)	5.7% (*n* = 9)	0.6% (*n* = 1)	23.9% (*n* = 38)	13.8% (*n* = 22)	0.0% (*n* = 0)	1.3% (*n* = 2)
Showing/Hacking (*n* = 69)	52.9% (*n* = 36)	1.4% (*n* = 1)	0.0% (*n* = 0)	18.9% (*n* = 14)	18.9% (*n* = 14)	1.4% (*n* = 1)	2.7% (*n* = 2)
Other (pooled remaining)(*n* = 141)	64.4% (*n* = 121)	6.9% (*n* = 13)	4.3% (*n* = 8)	11.2% (*n* = 21)	5.9% (*n* = 11)	1.1% (*n* = 2)	6.4% (*n* = 12)

**Table 5:** Respondents’ (*n* = 2323) reports of how often they tighten nosebands immediately before competition/racing. Each cell is the number of unique entries for each option in the survey which includes the percentage of responses out of the total (*n* = 2323). Respondents were able to select only one response. Their responses are categorised by type of noseband used.

Response	Noseband Type							
	Hanoverian	Sheepskin	Micklem	Figure-of-Eight/Grackle	Drop	Plain Cavesson	Other	Total
Always	0.7% (*n* = 4)	0	0.3% (*n* = 1)	0%	0.1% (*n* = 1)	0.6% (*n* = 7)	0	0.6% (*n* = 13)
Usually	1.7% (*n* = 10)	0	1.2% (*n* = 4)	4.3% (*n* = 3)	0.1% (*n* = 1)	1.1% (*n* = 12)	2.5% (*n* = 2)	1.4% (*n* = 32)
Sometimes	4.0% (*n* = 23)	10% (*n* = 1)	4.7% (*n* = 15)	10.0% (*n* = 7)	3.0% (*n* = 5)	2.4% (*n* = 26)	2.5% (*n* = 2)	3.4% (*n* = 79)
Rarely	10.4% (*n* = 60)	0	5.0% (*n* = 16)	4.3% (*n* = 3)	11.5% (*n* = 19)	5.9% (*n* = 64)	3.7% (*n* = 3)	7.1% (*n* = 165)
Never	83.2% (*n* = 480)	90% (*n* = 9)	88.8% (*n* = 285)	81.4% (*n* = 57)	84.2% (*n* = 139)	89.9% (*n* = 971)	91.4% (*n* = 74)	87.5% (*n* = 2015)
TOTAL	24.8% (*n* = 577)	0.4% (*n* = 10)	13.9% (*n* = 321)	3.0% (*n* = 70)	7.1% (*n* = 165)	46.9% (*n* = 1080)	3.8% (*n* = 81)	2323

**Table 6:** Frequency across disciplines of Anatomical, Consequential or Passive reasons for noseband use. Respondents (who used nosebands, *n* = 2332) were asked to select up to five responses from the list of reasons. Percentages reflect the percentage of respondents within a discipline who selected at least one reason from Anatomical, Consequential or Passive.

Discipline	Anatomical Reasons %	Consequential Reasons %	Passive Reasons %
Dressage (*n* = 800)	40.4	48.0	60.1
Eventing (*n* = 209)	49.8	59.3	59.3
Natural Horsemanship (*n* = 40)	25.0	38.9	58.3
Pleasure/Fun (*n* = 360)	31.2	38.4	53.9
Pony Club/Working Equitation (*n* =161)	42.8	57.2	54.1
Racing (*n* = 50)	60.8	70.6	25.5
Show-jumping (*n* = 264)	53.0	56.1	48.1
Showing/Hacking (*n* = 68)	50.0	60.3	57.4
Trail riding/Pleasure riding (*n* = 191)	30.9	35.1	55.2
Other (*n* = 141)	40.7	40.2	37.6

**Table 7:** Distribution across noseband types of Anatomical, Consequential or Passive reasons for noseband use (*n* = 2316).

Noseband Type	Anatomical Reasons %	Consequential Reasons %	Passive Reasons %
Sheepskin (*n* = 10)	10.0	20.0	50.0
Micklem (*n* = 322)	51.6	44.1	59.3
Hanoverian (*n* = 579)	62.0	56.0	50.8
Figure-of-Eight/Grackle (*n* = 72)	66.7	70.8	30.6
Drop (*n* = 165)	70.9	50.3	45.5
Plain Cavesson (*n* = 1087)	23.6	45.1	59.8
Other (*n* = 81)	11.1	34.6	30.9

**Table 8:** The distribution of complications reported by respondents who used nosebands, ranked from least number of responses to most. In total, 552 respondents reported one or more complications, with 939 individual complications recorded. The per cent of respondents who reported a complication is represented in the third column, with the per cent of the complication out of the total number of complications reported in the fourth column.

Complication	Number of Responses	% of Respondents (*n* = 434)	% of Responses (*n* = 939)
Reduced appetite	5	1.2	0.5
Bleeding from the mouth	20	4.6	2.1
Dropping food	33	7.6	3.5
Swelling of the area under the noseband	39	9.0	4.2
Discolouration of the area under the noseband	50	11.5	5.3
Head shyness	57	13.1	6.1
Soreness in the area under the noseband	62	14.3	6.6
Lip injuries	64	14.7	6.8
Behavioural signs of anxiety/distress	79	18.2	8.4
Difficulty bridling the horse	105	24.2	11.2
Other	124	28.6	13.2
Difficulty fastening the noseband	128	29.5	13.6
Hair loss in the area under the noseband	173	39.9	18.4

## Supplementary Materials

A full transcript of the questionnaire this report is based off is available online at https://www.mdpi.com/2076-2615/10/5/776/s1.
